# Cost-effectiveness of cervical cancer screening: cytology versus human papillomavirus DNA testing

**DOI:** 10.1111/j.1471-0528.2011.03228.x

**Published:** 2012-01-18

**Authors:** J van Rosmalen, IMCM de Kok, M van Ballegooijen

**Affiliations:** Department of Public Health, Erasmus MC, University Medical CenterRotterdam, the Netherlands

**Keywords:** Cervical cancer, cost-effectiveness analysis, HPV test, human papillomavirus, screening

## Abstract

**Objective:**

To determine the most cost-effective screening programme for cervical cancer.

**Design:**

Cost-effectiveness analysis from a societal perspective.

**Setting:**

The Netherlands.

**Population:**

Dutch women who have not been invited for human papillomavirus (HPV) vaccination.

**Methods:**

We calibrated the microsimulation screening analysis (MISCAN) model to Dutch epidemiological data. We used this model to consider nine screening strategies that use: (i) cytological testing with cytology triage for borderline/mildly abnormal smears; (ii) HPV testing with cytology triage for HPV-positive smears; or (iii) cytological testing with HPV triage for borderline/mildly abnormal smears. For each strategy, we varied the number of screening rounds, the time interval, the age of the first screening, and the type of cytological testing (conventional or liquid-based cytology).

**Main outcome measures:**

Quality-adjusted life years (QALYs) gained and costs from a societal perspective.

**Results:**

Under the base-case assumptions, primary HPV testing with cytology triage is the most cost-effective strategy. Using cost-effectiveness thresholds of €20 000 and €50 000 per QALY gained yields optimal screening programmes with three and seven screening rounds, respectively. The results are sensitive to several uncertain model inputs, most importantly the costs of the HPV test. For women aged 32 years or younger, primary cytology screening is more cost-effective than primary HPV testing.

**Conclusions:**

Increasing the interval between screening rounds and changing the primary test from cytology to HPV testing can improve the effectiveness and decrease the costs of cervical cancer screening in the Netherlands.

## Introduction

Cervical cancer mortality in the Netherlands has been steadily declining in the last decades, largely as a result of a well-functioning, cytology-based screening programme in which women are invited for a Pap smear every 5 years from age 30 to 60 years.^1^ However, the discovery of the human papillomavirus (HPV) as a necessary factor for developing cervical cancer has led to the introduction of HPV DNA testing and HPV vaccination,^2^ which may improve the health and economic outcomes of cervical cancer prevention.

Compared with cytology, the HPV DNA test has greater sensitivity but lower specificity in detecting high-grade cervical lesions.^3^ Results from randomised controlled trials have shown that HPV testing at baseline leads to a lower detection rate of cervical lesions at the next screening round than cytology-based screening.^4^–^7^ Another innovation is liquid-based cytology (LBC), which appears to have similar sensitivity and specificity as conventional cytology.^8^ However, LBC can decrease the proportion of inadequate smears,^9^ and enables us to perform a triage test (e.g. an HPV test) on the same material.

A final innovation is HPV vaccination, which can protect against the initial infection with HPV types 16 and 18, and, as a result, prevent the development of cervical dysplasia, and is thereby expected to prevent cervical cancer.^10^ In the Netherlands, 12-year-old girls are currently being invited for HPV vaccination; all women born after 1992 are eligible for HPV vaccination. However, the vaccinated girls will not reach the initial screening age (i.e. 30 years) until 2023, and millions of unvaccinated Dutch women born before 1993 will continue to be screened until they have reached the last screening age.

In this paper, we consider the cost-effectiveness of using only the HPV test as the primary test in the Netherlands, instead of cytology. This decision analysis is most relevant, because recently published data from randomised controlled trials enable us to better estimate the long-term effectiveness of the HPV test.^4^,^6^ We restrict our analysis to unvaccinated women. Determining the most cost-effective screening programme for vaccinated women will require a separate cost-effectiveness analysis that can be performed when more is known about the long-term effectiveness of HPV vaccination. The cost-effectiveness of HPV vaccination has been considered elsewhere.^11^–^16^

To determine the cost-effectiveness of adopting the HPV test as the primary test, it is not sufficient to compare screening programmes that only differ in the type of primary test. For example, compared with cytology, an HPV-based screening programme may require a longer interval between screening rounds, as a negative HPV test provides a longer duration of reduced risk.^17^ A positive primary HPV test may also require a different triage schedule than a positive primary cytology result. Therefore, we should compare the most efficient screening programmes with primary HPV testing with the most efficient cytology-based programmes.

Previous modelling studies have also analysed the cost-effectiveness of cervical screening in the Netherlands,^18^–^21^ and elsewhere.^22^–^32^ However, most of these studies did not consider a large number of alternative triage schedules and screening intensities. Also, some of these studies did not consider the HPV test as the only primary test.^18^,^26^–^30^

In the present study, we perform a comprehensive cost-effectiveness analysis of primary HPV testing versus primary cytology testing. We aim to find the most cost-effective combination of a screening strategy (i.e. the type of primary test and the triage schedule) and a screening policy (i.e. the ages at which women are invited for screening) for unvaccinated women in the Netherlands. We compare a variety of nationally and internationally recommended HPV and cytology triage schedules, using cost-effectiveness thresholds of €20 000 and €50 000 per quality-adjusted life year (QALY) gained. Finally, we perform a threshold analysis to investigate under what circumstances the HPV test is the most cost-effective primary test.

## Methods

We considered nine different screening strategies: (A) primary cytological testing with cytology triage for borderline/mildly abnormal smears; (B) primary HPV testing with a combination of cytology and HPV triage for HPV positive tests; (C, D, and E) primary HPV testing with cytology triage for HPV positive tests; (F, G, and H) primary cytological testing with a combination of cytology and HPV triage for borderline/mildly abnormal smears and (I) primary cytological testing with HPV triage for borderline/mildly abnormal smears (see Figure S1). These strategies are based on the literature,^21^,^33^–^36^ and include the current Dutch programme (strategy A). For all screening strategies, we considered a large number of screening policies using both conventional cytology and LBC.

### The MISCAN model

The costs and the effects of the different programmes were estimated using the microsimulation screening analysis (MISCAN) model.^12^,^20^,^37^ Some model assumptions were also used in our previous publications.^12^,^20^ In MISCAN, a large population is simulated that consists of hypothetical individual life histories, in which some women develop high-risk HPV infection(s), cervical neoplasia or cancer. This simulation yields an age-specific output of the prevalence of HPV infections and cervical neoplasia, and the incidence and the mortality of cervical cancer. This simulated population then undergoes simulated screening, which changes some of the life histories. The effects of screening can be determined from the changes in the life histories using the numbers of events and stages induced or prevented. This approach yields the changes in the number of life years, the quality of life and the costs.

### Model specifications: demography, epidemiology and natural history

The Dutch population at risk for cervical cancer was simulated based on demographic and hysterectomy data;^38^,^39^ mortality from other causes was estimated using the observed age-specific mortality in the Netherlands in 2008.^38^ The age-specific incidence of HPV infections that progress to cervical cancer was calibrated to the age distribution of the prescreening mortality in the Netherlands; the latter distribution was corrected for cohort effects based on an age–period–cohort analysis.^40^ The estimated cumulative incidence of HPV infections that progress to cervical cancer is 1.06% for women born in the period 1939–1948, and 1.48% for women born after 1948; these assumptions have been used previously.^12^ The age-specific incidence of preinvasive lesions that do not progress to cancer was calibrated so that the simulated detection rates of cervical intraepithelial neoplasia (CIN) fit the observed CIN detection rates in the Netherlands; the observed detection rates were obtained from the Dutch Network and National Database for Pathology (PALGA) for the period 1997–2001. Finally, the age-specific incidence of high-risk HPV infections that do not progress to CIN was calibrated so that the simulated prevalence of all high-risk HPV infections fits the observed high-risk HPV prevalence.^41^

In the model, the disease is subdivided into seven sequential stages: high-risk HPV infection (low-risk HPV infections are not simulated in the model); three pre-invasive stages (CIN 1, 2 and 3), and three invasive stages (International Federation of Obstetrics and Gynecology staging, FIGO 1A, 1B and 2+). Pre-invasive stages and FIGO 1A cases can only be diagnosed by screening, as they are assumed to be asymptomatic, whereas FIGO 1B and 2+ cases can also be clinically diagnosed. HPV infections are usually not progressive; in the model, more than 90% of HPV infections will clear without resulting in CIN, and most lesions in the pre-invasive stages regress naturally. For example, in the absence of cervical screening, approximately 72% of the CIN 3 lesions in the model would not become cancer, which corresponds well with estimates from a retrospective cohort study of women with untreated CIN 3.^42^ In a CIN lesion, a high-risk HPV infection may or may not be present. In the model it is assumed that, without an HPV infection, a CIN lesion will not progress to cervical cancer. In the invasive stages, all women are assumed to be HPV infected. A woman can develop multiple HPV infections and neoplasias in her lifetime, and multiple infections and neoplasias can exist at the same time. Weibull probability distributions are used to assume variation among women in the durations of the different stages. The inputs on stage-specific survival in clinical cases are age specific, and are based on observed survival data and on Dutch mortality-to-incidence ratios from the pre-screening period in the Netherlands.^40^

We used a population model that simulates the life histories of 8 million unvaccinated women born from 1939 to 1992. Women born before 1939 are too old to attend screening after 2011, and women born after 1992 are eligible for HPV vaccination. The simulated screening programmes start in 2011 and continue until all women have completed their screening programmes. Screening practices before 2011 can influence the effectiveness of the screening programme after 2011. Therefore, we also simulated the last three screening rounds before 2011, based on the assumption that earlier screening rounds (before 1996) will not affect the screening results after 2011. Information on the screening activities before 2011 was obtained from PALGA.

### Assumptions for screening and treatment

In our analyses, we varied the ages at which screening takes place. We considered all screening policies with starting ages of 25, 27, 30 or 32 years that comprise at least three and at most ten screenings in a woman’s lifetime, and that have an interval of at least 3 years and at most 10 years; policies that include screenings over the age of 70 years were not simulated. We considered both conventional cytology and LBC for each of the nine screening strategies in Figure S1. For the strategies that include a triage test immediately after the primary test, we considered two options: (i) co-collection of the material for the first triage test during the primary test and (ii) inviting women with a positive primary test for a separate triage test after 2 weeks. With option (i), the material for the first triage test is only evaluated if the result of the primary test requires a triage test. The co-collection of material for the triage test leads to a small increase in the cost of the primary test (see Table 2). For each strategy, we simulated all screening policies described above, with both conventional cytology and LBC, and with both options for collecting the material for the triage test.

We assumed that 10% of the population never attends screening and has a three times higher background risk (i.e. using the cervical cancer incidence in a situation without screening) than the 90% potential attenders. This assumption is based on an analysis of data from the start of organised screening.^43^ We assumed that the potential attenders attend 80% of all primary screenings, so that the overall attendance rate is 72%; under the base-case assumptions, follow-up screenings and referrals for colposcopy are always attended.

In the base-case analysis, we assumed that the sensitivity and the specificity of cytology are the same for conventional cytology and LBC.^8^ The sensitivity of the HPV test (the probability of a positive test result if an HPV infection is present) was estimated at 94%, and the sensitivity of cytology was assumed to be 40% for CIN 1, 50% for CIN 2, and 75% for CIN 3 and invasive cervical cancer (see Table 1).^21^ The specificity of the HPV test (probability of a negative test for women without HPV infections) is assumed to be 100%, and the specificity of cytology (probability of a negative test for women without CIN or cancer) is estimated to be 98.5%, based on the observed false-positive rate of Pap smears in the Dutch screening programme. Several screening strategies distinguish between smears read as ASC-US/LSIL (atypical squamous cells and low-grade cervical squamous intraepithelial lesions, equivalent to borderline/mild dyskaryosis) and smears read as at least HSIL (high-grade cervical squamous intraepithelial lesions, equivalent to moderate dyskaryosis). Therefore, the probability of at least HSIL is also specified for each disease stage in Table 1. The detection and the associated management (including retreatment, if necessary) of pre-invasive lesions were assumed to lead to a 100% cure rate. For screen-detected invasive cancers, the survival was modelled as a reduction in the risk of dying from cervical cancer compared with that of dying from clinically diagnosed cancer: in the model, detection by screening of an invasive cancer results in a reduction of the risk of dying of cervical cancer of 80% (FIGO 1A), 60% (FIGO 1B) or 20% (FIGO 2+).

**Table 1:** Assumptions for screening

	Parameter	Value
HPV screening	Attendance of potential attenders	80%
	Attendance of non-attenders	0%
	Sensitivity for high-risk HPV infection	94%
	Specificity for high-risk HPV infection	100%*
Cytological screening (conventional and LBC)	Attendance of potential attenders**	80%
	Sensitivity for CIN 1	40%
	Sensitivity for CIN 2	50%
	Sensitivity for CIN 3 and invasive cervical cancer	75%
	Probability of at least HSIL for CIN 1	4%
	Probability of at least HSIL for CIN 2	19%
	Probability of at least HSIL for CIN 3 and invasive cervical cancer	47%
	Specificity for at least CIN 1	98.5%

* Potential false-positive HPV test results were modelled as HPV infections with a short duration.

** The potential attenders consist of 90% of the female population; the remaining women are assumed to never attend screening.

### Assumptions for costs and utilities

Table 2 presents the costs and utilities used in the analysis. The estimated costs are based on a societal perspective, and are reported in 2010 euros (€). The screening costs include the costs for the invitational system and quality assurance, the time and travel costs of the woman being screened, the costs of smear taking, the costs of cytological evaluation, the costs of repeat tests after an inadequate test result and the costs of registration in PALGA. The laboratory costs of LBC were assumed to be €12 higher than for conventional cytology, mainly because of higher material and logistic costs.^44^ The diagnosis costs for women referred for colposcopy, the treatment costs for detected pre-invasive lesions, the costs of primary treatment for invasive cervical cancer, and the costs of treatment and palliative care for advanced cervical cancer were derived from previous cost studies performed in the Netherlands.^45^ In the model, a small loss of the quality of life is assumed for attending the primary screening test and for spending time in triage after a positive primary test. The loss in quality of life associated with a primary test comprises the time needed to attend the test and any anxiety caused by waiting for the result. Larger losses in quality of life are assumed for the diagnosis and treatment of CIN and cervical cancer, and for having a terminal disease. Utilities were based on (inter)nationally published data.^25^,^40^,^46^

**Table 2:** Base-case assumptions on costs, and level and duration of lost utility for different events and health states (costs are given in 2010 prices)

Type of test	Category	Costs (€)	Utilities lost	
	
			Level	Duration
	
	Invitation	4.74	0	–
Primary cytology*	Laboratory costs	21.77**	0.006	2 weeks
Organisation	11.23			
GP costs	11.76			
Time/travel	6.01			
Programme costs	2.08			
Repeat cytology	GP costs	22.21	0.006	Time since last test***
Laboratory costs	26.54**			
Time/travel	6.01			
Primary HPV test****	Laboratory costs	33.87	0.006	2 weeks
Organisation	11.23			
GP costs	11.76			
Time/travel	6.01			
Programme costs	2.08			
Repeat HPV test	GP costs	22.21	0.006	Time since last test***
Laboratory costs	33.87			
Time/travel	6.01			
Diagnosis and treatment pre-invasive stages	False positive referral	284	0.005	0.5 year
CIN 1	886	0.03	0.5 year	
CIN 2	1312	0.07	1 year	
CIN 3	1536	0.07	1 year	
Diagnosis and treatment invasive cancer	FIGO 1A	5031	0.062	5 years
FIGO 1B	11 930	0.062	5 years	
FIGO 2+ (detected by screening)	11 758	0.28	5 years	
FIGO 2+ (clinically detected)	10 982	0.28	5 years	
Terminal care	26 717	0.712	1 month	

* We assumed additional co-collection costs of €1 (for conventional cytology) or €0 (for LBC) to collect the material for a triage HPV test during a primary cytology test.

** For LBC, the costs are €33.72 for a primary test and €38.49 for a repeat test.

*** The time since the last test can be 0 weeks (in the case of co-collection during the primary test), 2 weeks (if a woman is invited for a repeat test immediately after a positive primary test), 6 or 12 months.

**** We assumed additional co-collection costs of €1 (conventional cytology) or €3 (for LBC) to collect the material for a triage cytology test during a primary HPV test.

### Cost-effectiveness analysis

The costs and the effects of each simulated screening programme are counted for the period from 2011 onwards. Future costs and health effects (life years and utility losses) are discounted in the base-case analysis towards the year 2011 at a rate of 3%. Programmes that are more costly and less effective than other programmes are ruled out as non-efficient (i.e. by simple dominance). Programmes that are more costly and less effective than a combination of other programmes are also ruled out as non-efficient (i.e. by extended dominance). The remaining programmes constitute the frontier of efficient screening programmes.

The total costs consist of the costs of the invitations (including the costs of the invitational system and the quality assurance), the primary and follow-up screenings, the treatment of pre-invasive and invasive lesions, and terminal care. We compute the net costs of screening as the difference in the total costs between the simulation in which the screening programme is implemented and a simulation without cervical screenings after 2011. The total number of QALYs is the number of years lived by the population minus the utility losses associated with attending screening, receiving treatment and having a terminal stage of cervical cancer. The number of QALYs gained by screening is the total number of QALYs in the simulation with the screening programme, minus the total number of QALYs in a simulation without screening after 2011.

### Sensitivity analyses

To assess the uncertainty in the model outcomes, we performed several sensitivity analyses. We investigate the consequences of varying the effectiveness criterion (life years gained instead of QALYs gained), the utility loss resulting from triage (no utility loss, two times higher utility loss and three times higher utility loss), the attendance at triage tests (among women invited for triage, 90% attend the triage tests instead of 100%), the laboratory costs of the HPV test (€20 and €45, instead of €33.87), the sensitivity of LBC compared with conventional cytology, the background risk for cervical cancer in screening attenders (30% higher than in the base-case analysis), and the type of discounting (1.5% for health effects and 4% for costs, instead of a uniform 3% rate). For the sensitivity analysis that explores a higher sensitivity of LBC, we increased the sensitivity of LBC for CIN and cervical cancer by five percentage points compared with the base-case analysis (e.g. 80% sensitivity for CIN 3 instead of 75%): this sensitivity analysis accounts for the fact that some studies have found a higher sensitivity for LBC than for conventional cytology.^47^,^48^ The sensitivity analysis with a higher background risk in screening attenders accounts for the possibility that the background risk does not differ between attenders and non-attenders (instead of a three times higher risk in non-attenders). This is because a decreased risk in non-attenders would have to be offset by an increased risk in attenders. Differential discounting using rates of 1.5% for health effects and 4% for costs is recommended by the Dutch Health Care Insurance Board,^49^ whereas a uniform rate of 3% is more common in international comparisons.

Among young women, because of the high prevalence of HPV infection, using a primary HPV test may lead to many unnecessary triage tests and colposcopies. Therefore, HPV testing may be less cost-effective for young women than for older women. We performed an additional analysis using screening strategy I (with primary cytology) for women younger than 33 years, and each of the four screening strategies with a primary HPV test for women aged 33 years or older.

Finally, in a threshold analysis, we investigate under what circumstances the HPV test is cost-effective as a primary test. Important variables affecting this decision are the laboratory costs of the HPV test and the utility loss resulting from spending time in triage; however, not many data are available on this utility loss, and the laboratory costs of the HPV test are negotiable and may change over time. We determine which values of these two variables would make primary HPV testing and primary cytology screening equally cost-effective.

## Results

**Figure 1:**
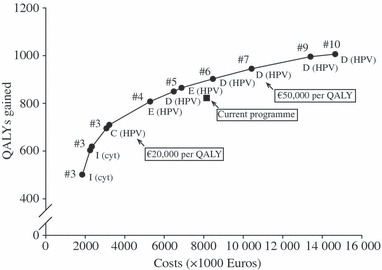
Net costs and health effects of the efficient screening programmes and the current screening programme in the Netherlands, for a cohort of 100 000 unvaccinated women, for the period from 2011 onwards. For each programme, the screening strategy and the number of scheduled examinations are shown. The arrows point to the current screening programme and the optimal programmes according to cost-effectiveness thresholds of €20 000 and €50 000 per QALY.

For the base-case analysis, the results of the programmes on the cost-effectiveness frontier are shown in Figure 1. This figure presents the expected future net costs and QALYs gained (from 2011 onwards) for a population of 100 000 women born from 1939 to 1992, for the remainder of their lives. More detailed results are presented in Tables S1 and S2.

All efficient screening programmes use strategies C or D (primary HPV screening with cytology triage), strategy E (primary HPV screening with a combination of cytology and HPV triage) or strategy I (primary cytology screening with HPV triage); however, strategy I was only cost-effective with a cost-effectiveness threshold below €7000 per QALY gained. All cost-effective programmes used conventional cytology instead of LBC. For strategies with a primary HPV test and cytology triage, collecting the material for the first triage test during the primary test is more cost-effective than letting women return for a triage test after 2 weeks. The incremental costs per QALY gained, which are obtained by comparing consecutive programmes on the efficient frontier, increase from €3701 for policies with three screening rounds to €122 508 for policies with ten screening rounds. At a cost-effectiveness threshold of €20 000 per QALY gained, three screening rounds during a woman’s lifetime with strategy C (primary HPV screening) is the optimal programme. Using a cost-effectiveness threshold of €50 000 per QALY gained yields a more intensive screening programme, with seven screening rounds.

The current Dutch screening programme is not among the most cost-effective programmes. For example, applying strategy D, using five screening rounds with a 6-year interval, starting at 30 years of age, yields more QALYs gained than the current programme at a 20% lower estimated cost.

### Sensitivity analyses

For each sensitivity analysis, Table 3 presents the efficient programme with an incremental cost-effectiveness ratio (ICER) just below cost-effectiveness thresholds of €50 000 per QALY gained; the results for the €20 000 per QALY threshold are shown in Table S3. In most sensitivity analyses, primary HPV testing using strategies C, D or E remains the most cost-effective option. These three strategies are, under most assumptions, approximately equally cost-effective. Assuming laboratory costs of the HPV test of €45 (instead of €33.87), or increasing the assumed utility loss associated with time spent in triage by a factor of three, would make strategy I (primary cytology with HPV triage) the most cost-effective strategy. Primary cytology screening (strategy I) is also the most cost-effective option if 10% of the women with a positive primary test do not attend the triage tests. Assuming a higher sensitivity of LBC, compared with conventional cytology, leads to the result that LBC is cost-effective only as a triage test after a primary HPV test. The optimal intensity of screening is sensitive to the cervical cancer risk in screening attenders, the cost-effectiveness threshold, and the rates at which costs and effects are discounted to the present. Finally, the results of the analysis with cytology screening for women younger than 33 years of age and HPV screening for older women show that this strategy is more cost-effective than screening programmes in which the primary test does not vary with age.

**Table 3:** Sensitivity analyses: efficient screening programmes with an ICER just below a €50 000 per QALY cost-effectiveness threshold, in various situations. All results are per 100 000 simulated women, discounted using a 3% rate for costs and effects, except for the sensitivity analysis using differential discounting.

Sensitivity analysis	Strategy (type of primary test)*	Number of screening rounds	Interval (years)	Age range (years)	QALYs gained	Net costs (× €1000)	ICER (euros per QALY gained)
Base case	D (HPV test)	7	6	30–66	944	10 418	46 566
Life years gained instead of QALYs gained**	C (HPV test)	7	6	30–66	943	10 666	44 970
No triage utility loss	C (HPV test)	7	6	30–66	981	10 666	46 022
Two times higher triage utility loss	D (HPV test)	7	6	30–66	930	10 418	47 620
Three times higher triage utility loss	I (cytology)	9	5	27–67	927	10 635	49 662
Lower attendance at triage tests (90 instead of 100%)	I (cytology)	8	4	30–58	887	9109	39 544
Lower costs HPV test (€20 instead of €33.87)	D (HPV test)	9	5	27–67	995	10 636	47 106
Higher costs HPV test (€45 instead of €33.87)	I (cytology)	9	5	27–67	926	10 583	49 483
Sensitivity of LBC 5% higher than of conventional cytology	D (HPV test)***	7	6	30–66	1002	10 412	46 299
Equal risk in screening attenders and non-attenders (30% higher background cervical cancer risk)	D (HPV test)	9	5	27–67	1304	12 598	46 277
Differential discounting (4% for costs, 1.5% for effects), €20 000 per QALY threshold****	E (HPV test)	5	6	30–54	1506	6523	15 539
Differential discounting (4% for costs, 1.5% for effects), €50 000 per QALY threshold****	D (HPV test)	9	5	27–67	1731	12 256	31 471
Cytology screening (strategy I) below 33 years of age, HPV screening for age 33 years and older	E (HPV test)	7	5	27–57	992	9297	40 384

* See Figure S1.

** For the analysis with life years gained, all results are calculated using life years gained instead of QALYs gained.

*** LBC is used as the triage test after a primary HPV test.

**** The analysis with differential discounting is presented for cost-effectiveness thresholds of €20 000 and €50 000 per QALY gained.

### Threshold analysis

In a threshold analysis, we investigated under which conditions the HPV test is the most cost-effective primary test. In the base-case analysis, we assumed laboratory costs of the HPV test of €33.87 and a utility loss per year spent in triage equivalent to 2.2 days of life. We find that primary cytology screening (strategy I) would become more cost-effective than primary HPV testing if the laboratory costs of the HPV test were above €42 (for the €20 000 per QALY cost-effectiveness threshold) or above €41 (for the €50 000 per QALY threshold). Increasing the utility loss resulting from spending time in triage from 2.2 days to at least 6 days per year would also make primary cytology screening (strategy I) the most cost-effective strategy.

## Discussion and conclusion

Despite the introduction of HPV 16/18 vaccination in the Netherlands and elsewhere, cervical screening will remain the most important cervical cancer prevention method for the majority of women in the coming decades. Modern alternatives (i.e. the HPV test and LBC) to conventional cytology may improve the efficiency of cervical cancer screening. This article has presented a large microsimulation study to evaluate the cost-effectiveness of the available cervical cancer screening strategies: we focused on the cost-effectiveness of adopting the HPV test as the primary test. Our analysis should help determine the optimal screening programme in the Netherlands and in other countries as well.

We found that primary HPV screening with cytology triage is the most cost-effective screening strategy under the base-case assumptions. In the base-case analysis, the optimal programme consists of HPV screening with cytology triage with three (at a €20 000 per QALY cost-effectiveness threshold) or seven (at a €50 000 per QALY cost-effectiveness threshold) screening rounds in a woman’s lifetime. However, an additional analysis showed that primary HPV screening is not cost-effective for young women: a combination of primary cytology screening for women younger than 33 years of age and HPV screening for older women can be more cost-effective than a programme with HPV screening for women of all ages. This result arises from the relatively low positive predictive value of HPV testing for progressive cervical lesions among younger women. All cost-effective screening programmes in this analysis used conventional cytology instead of LBC: the advantages of LBC (e.g. the possibility to perform an HPV test on the same material) did not outweigh the higher evaluation costs.

Sensitivity analyses have shown that several model inputs can affect what the optimal screening programme is. The optimal number of screening rounds is sensitive to the background cervical cancer risk in women attending screening, to the discount rates used for costs and health effects and to the cost-effectiveness threshold. An increase in the laboratory costs of the HPV test (from €34 to €42) or an increase in the utility loss associated with time spent in triage (from 2.2 to 6 days of life per year spent in triage) would make primary cytology more cost-effective than primary HPV testing. The latter result arises from the fact that strategy I (primary cytology screening with immediate HPV triage) does not require waiting for triage tests, whereas the strategies with primary HPV testing usually require 6 or 18 months of follow-up after a positive primary test. Assuming a somewhat higher sensitivity of LBC compared with conventional cytology, as found in previous studies,^47^,^48^ could make LBC more cost-effective than conventional cytology; however, the HPV test would still be the most cost-effective primary test in that case.

Although HPV testing has been possible for many years, only recent evidence from randomised controlled trials in the Netherlands, the UK, Italy and Sweden has shown that HPV testing at baseline leads to a lower detection rate of cervical lesions at the next screening round than cytology-based screening.^4^–^7^ Because of the higher sensitivity of the HPV test for CIN and cervical cancer, using this test as the primary test can yield more health effects than a cytology-based programme, even with fewer screening rounds. Nevertheless, the HPV test has some disadvantages. Because of the lower specificity of the HPV test, many women who do not have CIN or cancer attend triage tests after a positive HPV test. The uncertainty caused by waiting for a diagnosis after a positive test could cause anxiety. In addition, the HPV test may not be cost-effective if the ratio of the prevalence of HPV infections to the prevalence of CIN is high. This may be the case in some countries, e.g. in Italy,^50^,^51^ and in young women.^52^ Finally, the HPV test is currently more expensive than cytology, although this may change in the future.

The MISCAN model and the assumptions for costs and utilities were based on the Dutch situation. For other countries, the optimal screening intensity in particular can differ, based on the background risk of cervical cancer and other factors: for example, countries with a higher background level of cervical cancer incidence, e.g. Denmark,^53^ may require more screening rounds than the Netherlands. Nevertheless, some results presented here can be generalised to other countries. The optimal primary test and triage schedule will probably be the same as in our analysis for countries that have similar treatment and testing costs, and a similar positive predictive value of HPV testing for cancer and for CIN that would eventually become cancer. The latter condition requires that the ratio of the prevalence of HPV infection to the incidence of cervical cancer is approximately the same as in the Netherlands: this condition appears to be met in most countries.^54^ Also, the results of sensitivity analyses showed that, under varying circumstances, only strategies C, D and E (all primary HPV screening) or strategy I (primary cytology) were among the most cost-effective ones. In most of these strategies, women are only referred for colposcopy after a positive HPV test and a positive cytology result in the same screening round, or a cytological result of at least HSIL. These screening strategies may provide an optimal combination of the high sensitivity of the HPV test and the high specificity of cytology.

Several cost-effectiveness analyses of cervical cancer screening have been published previously.^18^–^32^ The analyses show a large variation in terms of what programmes are evaluated, the model assumptions and the methodological approach (e.g. the type of model). The results of our analysis suggest fewer screening rounds than in previous analyses. Reasons for this are that the background cervical cancer incidence in the Netherlands is lower than in most other countries, and that we assumed that women attending screening have a lower background risk than non-attenders. Most of the previous cost-effectiveness analyses support introducing the HPV test as a primary test. For example, a cost-effectiveness analysis of HPV testing in the Netherlands found that primary HPV screening can be more cost-effective than the current screening programme in the Netherlands.^21^ In that study, it was also suggested to increase the interval between screening rounds if the HPV test is used as the primary test. In one analysis that did not support introducing the HPV test, women were directly referred for colposcopy after a positive primary HPV test.^25^ Our analysis shows the importance of specifying an effective triage schedule for women with a positive primary test, as referring all women with a positive primary HPV test will result in a large number of unnecessary colposcopies. Another cost-effectiveness analysis suggested a programme of primary cytology with HPV triage (strategy I), with an interval of 3 years for the Netherlands;^18^ however, using only the HPV test as a primary test was not considered in that analysis. The main contribution of the present study is that it is more comprehensive than previous cost-effectiveness analyses of cervical cancer, with a much larger number of evaluated screening programmes. To provide a fair comparison between cytology and the HPV test, screening programmes with different numbers of screening rounds and triage schedules must be considered. We have also shown the effects of different assumptions for costs and utilities, different discount rates, and assuming a loss to follow-up.

Like any study, this study has some limitations. First, the cost-effectiveness results are sensitive to several model inputs. Some of these model inputs (e.g. the natural history of the disease) are not known exactly, whereas other inputs can change over time (e.g. the relative costs of cytology and HPV testing). Another limitation is that we did not take HPV 16/18 vaccination into account. Although the present analysis is exclusively intended for the unvaccinated cohorts, the vaccination of younger women may yield a herd immunity effect that can lower the incidence of cervical cancer in unvaccinated women. This effect could further reduce the optimal intensity of screening. However, we believe that any such effect is likely to be small, as there is a large age difference between the vaccinated women and the unvaccinated women considered here. Future research should evaluate screening in HPV-vaccinated women.

In summary, this paper has presented a comprehensive cost-effectiveness analysis of adopting the HPV test as the primary cervical screening test in the Netherlands. By comparing a variety of screening strategies and policies, we have shown how to optimally combine the type of screening test, the triage schedule, the screening age range and the intensity of screening. Our results support introducing the HPV test as the primary test in the Netherlands and increasing the interval between screening rounds. We have also shown the cost and utility conditions for which the HPV test is most cost-effective as a primary test.

## Supporting Information

**Figure S1.** Screening strategies evaluated inthe cost-effectiveness analysis: +, positive HPV test; –,negative HPV test; Colp, referral for colposcopy; Cyt, indicatescytology; HPV, HPV DNA testing; *t* = 0, time ofprimary test; *t* = 6 and *t* = 18indicates 6 and 18 months after the primary test,respectively.

**Table S1.** Efficient screening programmes,characterised by the screening strategy, the number of scheduledexaminations, the screening interval and the age range thatmaximise the number of QALYs gained (discounted at 3%) relative tothe costs (discounted at 3%). For each policy, the changes in costsand effects per 100 000 women and the ICER are given.

**Table S2.** Undiscounted effects per 100 000simulated women in the efficient screening programmes shown inTable S1: total effects, from 2011 onwards, for the remainder ofthe lives of the simulated women.

**Table S3.** Sensitivity analyses: efficientscreening programmes with ICERs just below the €20 000and €50 000 per QALY cost-effectiveness thresholds, invarious situations. All results are per 100 000 simulatedwomen, discounted using a 3% rate for costs and effects, except forthe sensitivity analysis using differential disco.
