# Supplementary material for: Cost-effectiveness of cervical cancer screening: cytology versus human papillomavirus DNA testing
Source: BJOG. 2012 Jan 18;119(6):699–709. doi: 10.1111/j.1471-0528.2011.03228.x (PMC3489039; doi:10.1111/j.1471-0528.2011.03228.x)
Supplement: Supplementary file 2 [file bjo0119-0699-SD2.pdf]

**Table S1.** Efficient screening programmes, characterised by the screening strategy, the number of scheduled examinations, the screening interval, and the age range that maximize the number of quality adjusted life years (QALYs) gained (discounted at 3%) relative to the costs (discounted at 3%). For each policy, the changes in costs and effects per 100,000 women and the incremental cost-effectiveness ratio (ICER) are given.

| Strategy[1] (type of primary test) | Number of screening rounds | Interval (years) | Age range | Co-collection | Type of cytology | QALYs gained | Net costs (x 1000 euros) | ICER (euros per QALY gained) |
|---|---|---|---|---|---|---|---|---|
| I (cytology) | 3 | 8 | 25-41 | No | Conventional | 501 | 1,854 | 3,701 |
| I (cytology) | 3 | 5 | 32-42 | No | Conventional | 603 | 2,256 | 3,945 |
| I (cytology) | 3 | 6 | 32-44 | No | Conventional | 618 | 2,356 | 6,606 |
| C (HPV test) | 3 | 7 | 30-44 | Yes | Conventional | 695 | 3,090 | 9,558 |
| C (HPV test) | 3 | 6 | 32-44 | Yes | Conventional | 709 | 3,233 | 10,300 |
| E (HPV test) | 4 | 8 | 30-54 | Yes | Conventional | 806 | 5,284 | 21,130 |
| D (HPV test) | 5 | 6 | 30-54 | Yes | Conventional | 849 | 6,480 | 27,354 |
| E (HPV test) | 5 | 6 | 30-54 | Yes | Conventional | 864 | 6,871 | 27,739 |
| D (HPV test) | 6 | 6 | 30-60 | Yes | Conventional | 902 | 8,472 | 41,859 |
| D (HPV test) | 7 | 6 | 30-66 | Yes | Conventional | 944 | 10,418 | 46,566 |
| D (HPV test) | 9 | 5 | 27-67 | Yes | Conventional | 995 | 13,398 | 57,514 |
| D (HPV test) | 10 | 5 | 25-70 | Yes | Conventional | 1,006 | 14,648 | 122,508 |
| A (cytology, current programme) | 7 | 5 | 30-60 | No | Conventional | 821 | 8,149 | |

[1]See Figure S1
