# Supplementary material for: Cost-effectiveness of cervical cancer screening: cytology versus human papillomavirus DNA testing
Source: BJOG. 2012 Jan 18;119(6):699–709. doi: 10.1111/j.1471-0528.2011.03228.x (PMC3489039; doi:10.1111/j.1471-0528.2011.03228.x)
Supplement: Supplementary file 3 [file bjo0119-0699-SD3.pdf]

Table S2. Undiscounted effects per 100,000 simulated women of the efficient screening programmes shown in Table S1. This table presents total effects, from 2011 onwards, for the remainder of the lives of the simulated women.

Strategy[1] (type of primary test)	Number of screening rounds	Interval (years)	Age range	Primary screenings	Positive primary screenings	Triage screenings	False positive referrals	Screen-detected CIN 2 or CIN 3 lesions	Screen-detected cases of cancer	Clinically detected cases of cancer	Deaths from cervical cancer	Life years lost	QALYs lost
I (cytology)	3	8	25-41	53,835	1,528	1,191	75	452	22	393	208	6,137	6,603
I (cytology)	3	5	32-42	68,094	1,866	1,471	79	526	25	367	197	5,848	6,298
I (cytology)	3	6	32-44	71,954	1,948	1,543	81	537	26	363	194	5,795	6,243
C (HPV test)	3	7	30-44	68,394	3,845	10,104	132	634	27	339	182	5,495	5,955
C (HPV test)	3	6	32-44	71,991	4,016	10,599	138	638	28	334	181	5,461	5,920
E (HPV test)	4	8	30-54	115,261	4,960	9,214	1,603	751	35	306	162	5,161	5,608
D (HPV test)	5	6	30-54	144,319	5,952	11,082	142	786	35	297	160	5,075	5,509
E (HPV test)	5	6	30-54	144,459	5,879	10,976	1,926	817	34	290	156	4,990	5,439
D (HPV test)	6	6	30-60	192,378	6,971	13,017	168	872	38	277	148	4,906	5,338
D (HPV test)	7	6	30-66	243,837	7,652	14,281	184	963	41	255	135	4,772	5,202
D (HPV test)	9	5	27-67	302,434	10,781	20,387	265	1,048	39	239	127	4,595	5,038
D (HPV test)	10	5	25-70	335,979	10,704	20,180	263	1,103	40	229	121	4,552	4,993
A (cytology, current programme)	7	5	30-60	224,535	4,968	7,963	221	860	39	290	154	5,075	5,546

[1]See Figure S1
