# Supplementary material for: Cost-effectiveness of cervical cancer screening: cytology versus human papillomavirus DNA testing
Source: BJOG. 2012 Jan 18;119(6):699–709. doi: 10.1111/j.1471-0528.2011.03228.x (PMC3489039; doi:10.1111/j.1471-0528.2011.03228.x)
Supplement: Supplementary file 1 [file bjo0119-0699-SD1.pdf]

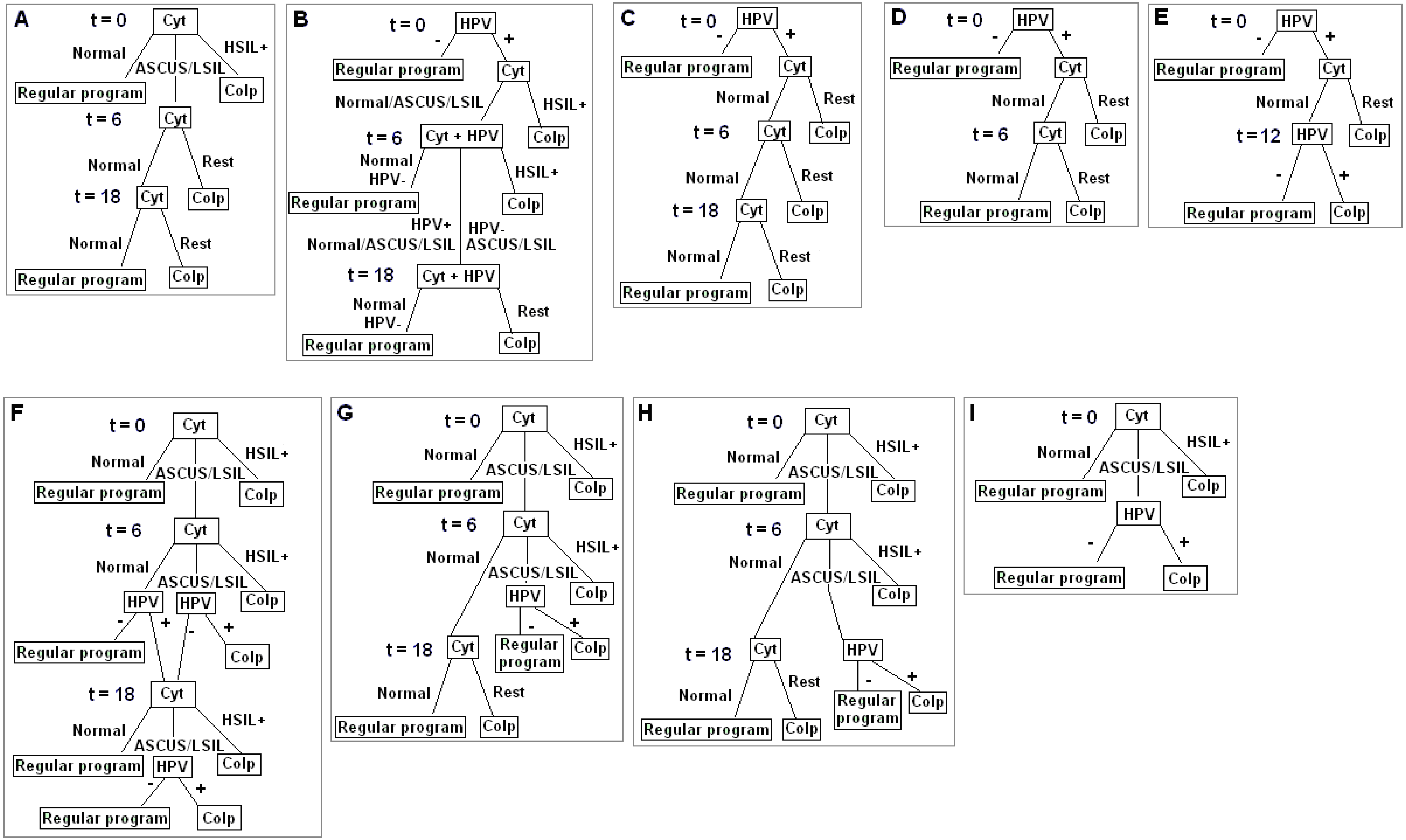

Figure S1. Screening strategies evaluated in the cost-effectiveness analysis. HPV indicates HPV DNA testing, Cyt indicates cytology, + indicates a positive HPV test, - indicates a negative HPV test, Colp indicates referral for colposcopy, t = 0 indicates the moment of primary test, t = 6 and t = 18 indicates 6 and 18 months after the primary test, respectively.
