# Supplementary material for: Cost-effectiveness of cervical cancer screening: cytology versus human papillomavirus DNA testing
Source: BJOG. 2012 Jan 18;119(6):699–709. doi: 10.1111/j.1471-0528.2011.03228.x (PMC3489039; doi:10.1111/j.1471-0528.2011.03228.x)
Supplement: Supplementary file 4 [file bjo0119-0699-SD4.pdf]

Table S3. Sensitivity analyses: efficient screening programmes with an incremental cost-effectiveness ratio (ICER) just below a €20,000 and a €50,000 per QALY cost-effectiveness threshold, in various situations. All results are per 100,000 simulated women, discounted using a 3% rate for costs and effects, except for the sensitivity analysis using differential discounting.

Sensitivity analysis	Cost-effectiveness threshold	Strategy (type of primary test)[1]	Number of screening rounds	Interval (years)	Age range	QALYs gained	Net costs (x 1000 euros)	ICER (euros per QALY gained) [2]
Base case	€20,000	C (HPV test)	3	6	32-44	709	3,233	10,300
	€50,000	D (HPV test)	7	6	30-66	944	10,418	46,566
Life years gained	€20,000	C (HPV test)	3	6	32-44	691	3,233	10,643
	€50,000	C (HPV test)	7	6	30-66	943	10,666	44,970
No triage utility loss	€20,000	C (HPV test)	3	6	32-44	731	3,233	9,721
	€50,000	C (HPV test)	7	6	30-66	981	10,666	46,022
Two times higher triage utility loss	€20,000	D (HPV test)	4	6	30-48	761	4,574	19,674
	€50,000	D (HPV test)	7	6	30-66	930	10,418	47,620
Three times higher triage utility loss	€20,000	I (cytology)	4	5	32-47	707	3,597	17,186
	€50,000	I (cytology)	9	5	27-67	927	10,635	49,662
Lower attendance at triage tests (90% instead of 100%)	€20,000	I (cytology)	5	4	30-46	735	4,406	18,769
	€50,000	I (cytology)	8	4	30-58	887	9,109	39,544
Lower costs HPV test (€20 instead of €33.87)	€20,000	E (HPV test)	4	8	30-54	806	4,099	16,771
	€50,000	D (HPV test)	9	5	27-67	995	10,636	47,106
Higher costs HPV test (€45 instead of €33.87)	€20,000	I (cytology)	4	5	32-47	708	3,615	13,901
	€50,000	I (cytology)	9	5	27-67	926	10,583	49,483
Sensitivity of LBC 5% higher than of conventional cytology	€20,000	D (HPV test)[3]	3	6	32-44	764	4,055	8,990
	€50,000	D (HPV test)[3]	7	6	30-66	1,002	10,412	46,299
Equal risk in screening attenders and non-attenders (30% higher background cervical cancer risk)	€20,000	C (HPV test)	4	8	32-56	1,061	4,822	15,188
	€50,000	D (HPV test)	9	5	27-67	1,304	12,598	46,277
Differential discounting (4% for costs, 1.5% for effects)	€20,000	E (HPV test)	5	6	30-54	1,506	6,523	15,539
	€50,000	D (HPV test)	9	5	27-67	1,731	12,256	31,471
Cytology screening below age 33 (strategy I), HPV screening for ages 33 and older	€20,000	E (HPV test)[5]	3	6	32-44	778	2,981	11,520
	€50,000	E (HPV test)[5]	7	5	27-57	992	9,297	40,384

[1] See Figure S1.

[2] For the analysis with life years gained, the ICER is given per life year gained.

[3] LBC is used as the triage test after a primary HPV test.
